# The clinical impact of mRNA therapeutics in the treatment of cancers, infections, genetic disorders, and autoimmune diseases

**DOI:** 10.1016/j.heliyon.2024.e26971

**Published:** 2024-02-29

**Authors:** Roham Deyhimfar, Mehrnaz Izady, Mohammadreza Shoghi, Mohammad Hossein Kazazi, Zahra Fakhraei Ghazvini, Hojjatollah Nazari, Zahra Fekrirad, Ehsan Arefian

**Affiliations:** a Department of Stem Cells Technology and Tissue Regeneration, School of Biology, College of Science, University of Tehran, Tehran, Iran; b Urology Research Center, Sina Hospital, Tehran University of Medical Sciences, Tehran, Iran; c Department of Biology, University of Copenhagen, Copenhagen, Denmark; d Department of Biomedical Sciences, Ontario Veterinary College, University of Guelph, ON, Canada; e Department of Animal Biology, School of Biology, College of Science, University of Tehran, Tehran, Iran; f School of Biomedical Engineering, University of Technology Sydney, Sydney, Australia; g Department of Biology, Faculty of Basic Sciences, Shahed University, Tehran, Iran; h Department of Microbiology, School of Biology, College of Science, University of Tehran, Tehran, Iran; i Pediatric Cell and Gene Therapy Research Center, Gene, Cell & Tissue Research Institute, Tehran University of Medical Sciences, Tehran, Iran

**Keywords:** mRNA therapy, Vaccine, Cancer, Genetic disorders, Autoimmune diseases

## Abstract

mRNA-based therapeutics have revolutionized medicine and the pharmaceutical industry. The recent progress in the optimization and formulation of mRNAs has led to the development of a new therapeutic platform with a broad range of applications. With a growing body of evidence supporting the use of mRNA-based drugs for precision medicine and personalized treatments, including cancer immunotherapy, genetic disorders, and autoimmune diseases, this emerging technology offers a rapidly expanding category of therapeutic options. Furthermore, the development and deployment of mRNA vaccines have facilitated a prompt and flexible response to medical emergencies, exemplified by the COVID-19 outbreak. The establishment of stable and safe mRNA molecules carried by efficient delivery systems is now available through recent advances in molecular biology and nanotechnology. This review aims to elucidate the advancements in the clinical applications of mRNAs for addressing significant health-related challenges such as cancer, autoimmune diseases, genetic disorders, and infections and provide insights into the efficacy and safety of mRNA therapeutics in recent clinical trials.

## Graphical abstract

**Figure:**
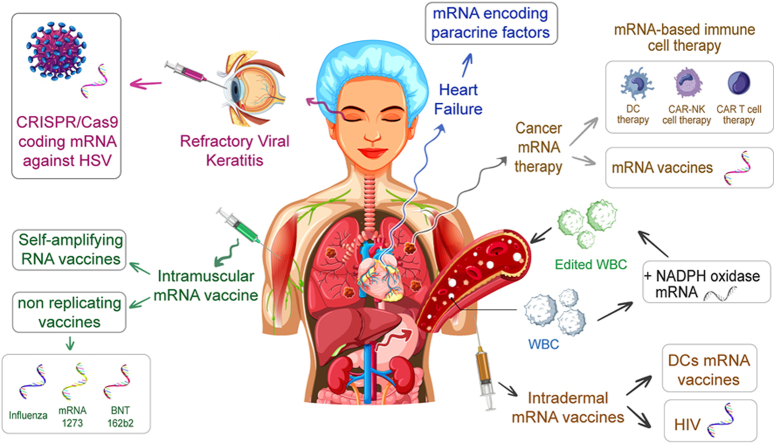

## 1 Introduction

Over recent years, messenger ribonucleic acid (mRNA)-based therapeutics have emerged as a promising form of medicine, revolutionizing gene therapy by introducing anticancer agents, vaccines, and immune-modulatory drugs. Research has shown that mRNAs possess a multitude of therapeutic advantages over other functional biomolecules including recombinant proteins and plasmid DNA. This is largely due to the high safety profile of mRNAs, as they do not integrate into the genome and cause insertional mutagenesis [1].

Exogenous mRNAs are mainly manufactured by In vitro transcription (IVT) technology, a process in which linearized DNA originating from a bacterial plasmid or polymerase chain reaction (PCR) product is utilized as the template for RNA polymerases such as T7, T3, and SP6 to produce mRNA of interest in a cell-free system [2]. Despite the IVT platform's industrial scalability, low cost, and astonishingly short design-to-release period, there are major challenges related to mRNA purity, stability, and delivery. In recent years, purification techniques such as high-performance liquid chromatography have been harnessed to decrease the presence of impurities such as double-stranded RNA, which can lead to unintended inflammation and subsequent immune responses [3]. The mRNA molecules have also been modified by utilizing modified nucleotides, adding a 5′ cap, and extending their poly-A tail to reduce their self-immunogenicity as well as increase their intracellular stability and translational efficiency. Moreover, naked mRNAs are extremely susceptible to RNAse-mediated degradation and are inefficiently taken up by the cell due to their large size and negative charge [4]. In the formulation process, there has been considerable progress in improving the delivery, extracellular stability, and storage/transport conditions of therapeutic mRNAs (without requiring cold-chain transportation) [5,6].

The field of mRNA-based drugs is undergoing a rapid transformation, significantly altering the standard of care for numerous diseases. The inherent flexibility in design and cost-effective mass production are significant advantages over traditional methods. Of particular note, mRNA vaccines have emerged as a promising strategy in the fight against infectious agents, such as the Coronavirus. The mRNA vaccines against SARS-CoV-2 are the first authorized mRNA vaccines that begin a new era in preventive medicine. Recent preclinical and clinical studies have provided encouraging results, prompting scientists to investigate novel applications of mRNAs for the treatment of various diseases. This article aims to review recent mRNA studies in the clinic and explore the impact and effectiveness of these emerging modern platforms.

## 2 mRNA therapeutics in cancer treatment

**Fig. 1:**
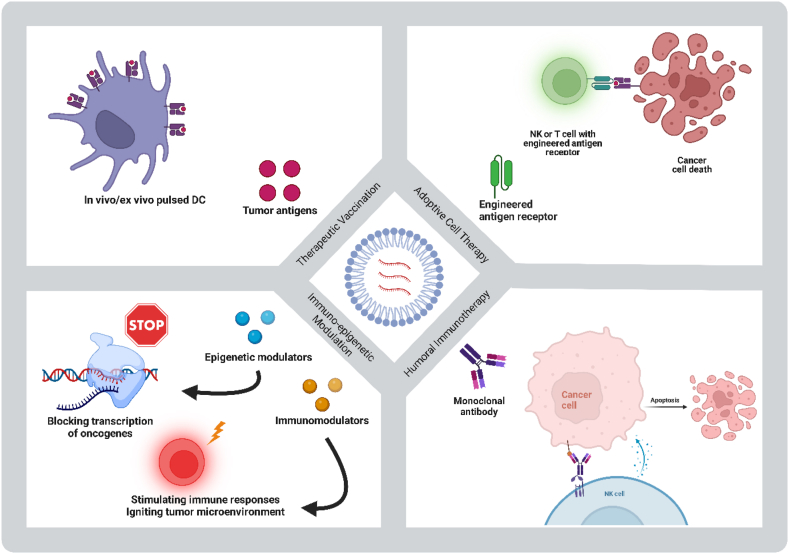
mRNA-based strategies for treatment of cancers.

Despite numerous advances in diagnosis and treatment, cancer is still considered one of the most significant challenges to human health. According to Global Cancer Statistics, in 2020, about 19.3 million new cases were diagnosed, and approximately 10 million people died of cancer in countries around the world [7]. mRNA technology platform has opened up new avenues for either novel therapies or optimization of standard treatments throughout the last two decades. The Flexible nature of mRNA technology can support a wide range of anti-cancer treatments, ranging from tumoral antigens to monoclonal antibodies and immunomodulators. In addition, patient-specific variations have richly colored the role of precision oncology in tailoring therapeutic modalities. Its manageability, cost-effectiveness, and rapid development place the mRNA platform in a superior position compared to its rivals. It should be noted that despite these great advantages, RNA-based therapeutics, including mRNAs and miRNAs, require formulation and modifications due to their instability and sensitivity. These factors can lead to adverse events that limit their efficacy and hinder the development process [8]. mRNA-based therapeutic strategies, which have been used clinically to confront cancer, can fall into four categories; therapeutic vaccination, adoptive cell therapy, humoral immunotherapy, and immuno-epigenetic modulation (Fig. 1). All of these strategies are now being tested in clinical studies, showing encouraging results (Table 1).

**Table 1:** List of all conducted clinical trials for mRNA-based treatment of cancers (Until Jan 2024).

	NCT Number	mRNA Drug Description (Name)	Delivery Method	Indication	Interventions	Trial Phase (Study Start Date)	Estimated Enrollments	Eligible Ages	Sponsor (Collaborators)	Locations	Status
Therapeutic Vaccination											
1	NCT05660408	Tumor mRNA Loaded Lipid Particles (RNA-LP)	In vivo	Pulmonary Osteosarcoma	Multiple-Dose Monotherapy	Phase I/II (2024)	43	Children and Adults (0–39)	University of Florida in collaboration of The V Foundation for Cancer Research	United States	Not yet recruiting
2	NCT05264974	Autologous Total Tumor mRNA Loaded DOTAP Liposome Vaccine (mRNA-NP)	In vivo	Melanoma	Multiple-Dose Monotherapy	Phase I (2024)	18	Adults (18–99)	University of Florida	United States	Recruiting
3	NCT06156267	Neoantigen Personalized mRNA Tumour Vaccine	In vivo	Pancreatic Cancer	Combination with Immune Checkpoint Inhibitors	Phase I (2024)	30	Adults (18–75)	Fudan University in collaboration of Shanghai Regenelead Therapies Co., Ltd.	China	Not yet recruiting
4	NCT06195384	Neoantigen mRNA Vaccine	In vivo	Solid Tumors	Monotherapy	Phase I (2024)	30	Adults (18–75)	Second Affiliated Hospital of Guangzhou Medical University	China	Not yet recruiting
5	NCT06141369	Individualized Neoantigen Vaccine (mRNA-0523-L001)	In vivo	Adrenal Cortical Carcinoma Medullary Thyroid Cancer Thymic Neuroendocrine Carcinoma Pancreatic Neuroendocrine Tumor	Multiple-Dose Monotherapy	N/A (2023)	21	Adults (≥18)	Shanghai Jiao Tong University School of Medicine	China	Not yet recruiting
6	NCT05799612	mRNA plus Lysate-Loaded Dendritic Cell Vaccine	Ex vivo	Cutaneous Angiosarcoma	Combination with Chemotherapy, Interferon alpha-2A, and Filgrastim	Phase 1 (2023)	24	Adults (≥18)	M.D. Anderson Cancer Center with Collaboration of Cancer Cures 4 Kids	United States	Not yet recruiting
7	NCT06019702	Encodes Personalized Neoantigens (iNeo-Vac-R01)	In vivo	Digestive System Neoplasms	Multiple-Dose Monotherapy	Phase 1 (2023)	20	Adults (18–75)	Sir Run Run Shaw Hospital with Collaboration of Hangzhou Neoantigen Therapeutics Co., Ltd.	China	Recruiting
8	NCT06026800	Encodes Personalized Neoantigens (iNeo-Vac-R01)	In vivo	Digestive System Neoplasms	Multiple-Dose Monotherapy Combination with Standard Adjuvant Therapy	Phase 1 (2023)	20	Adults (18–75)	Sir Run Run Shaw Hospital with Collaboration of Hangzhou Neoantigen Therapeutics Co., Ltd.	China	Recruiting
9	NCT06026774	Encodes Personalized Neoantigens (iNeo-Vac-R01)	In vivo	Digestive System Neoplasms	Multiple-Dose Monotherapy Combination with Standard Adjuvant Therapy	Phase 1 (2023)	20	Adults (18–75)	Sir Run Run Shaw Hospital with Collaboration of Hangzhou Neoantigen Therapeutics Co., Ltd.	China	Recruiting
10	NCT04741984	monocytes isolated from patient's leukapheresis loaded with CMV pp65-LAMP (Lysosomal-associated Membrane Protein) mRNA	Ex vivo	Glioblastoma	Multiple-Dose Monotherapy	Phase 1 (2023)	0	Adults (≥18)	Michael Gunn with Collaboration of National Cancer Institute (NCI)	United States	WITHDRAWN
11	NCT05981066	Encodes Neoantigen (ABOR2014/IPM511)	In vivo	Advanced Hepatocellular Carcinoma	Multiple-Dose Monotherapy	N/A (2023)	48	Adults (18–75)	Peking Union Medical College Hospital	China	Recruiting
12	NCT05942378	Encodes Neoantigens (HRXG-K-1939)	In vivo	Advanced Solid Tumors	Combination with Immune Checkpoint Inhibitors	Phase 1 (2023)	30	Adults (18–75)	Fudan University	China	Not yet recruiting
13	NCT05949775	Encodes Personalized Neoantigens	In vivo	Advanced Malignant Solid Tumors	Combination with Immune Checkpoint Inhibitors	N/A (2023)	20	Adults (≥18)	Stemirna Therapeutics with Collaboration of Peking University Cancer Hospital & Institute	China	Not yet recruiting
14	NCT05938387	Encodes GBM Peptides (CV09050101/CVGBM)	In vivo	Glioblastoma Astrocytoma	Multiple-Dose Monotherapy	Phase 1 (2023)	54	Adults (≥18)	CureVac	Germany Netherlands	Recruiting
15	NCT05761717	Encodes Neonatal Antigens	In vivo	Hepatocellular Carcinoma	Combination with Immune Checkpoint Inhibitors	N/A (2023)	67	Adults (≥18)	Shanghai Zhongshan Hospital	China	Not yet recruiting
16	NCT05940181	Encodes Neoantigens (XH001)	In vivo	Solid Tumors	Combination with Immune Checkpoint Inhibitors	N/A (2023)	9	Adults (18–75)	Jianming xu with Collaboration of NeoCura	China	Recruiting
17	NCT05738447	Encodes HVB Antigens	In vivo	Hepatocellular Carcinoma	Multiple-Dose Monotherapy	Phase 1 (2023)	9	Adults (18–70)	West China Hospital	China	Recruiting
18	NCT05579275	Encodes Neoantigens (JCXH-212)	In vivo	Advanced Malignant Solid Tumors	Monotherapy	Phase 1 (2023)	24	Adults (18–75)	Peking University Cancer Hospital & Institute	China	Recruiting
19	NCT05714748	Encodes EBV Antigens	In vivo	EBV-positive Advanced Malignant Tumors	Multiple-Dose Monotherapy	Phase 1 (2022)	9	Adults (18–70)	West China Hospital	China	Recruiting
20	NCT05359354	Encodes Personalized Neoantigens (PGV002)	In vivo	Advanced Solid Tumors	Monotherapy Combination with Immune Checkpoint Inhibitors	N/A (2022)	36	Adults (18–75)	YueJuan Cheng with Collaboration of NeoCura	China	Recruiting
21	NCT05264974	Autologous Whole Tumor mRNA	In vivo	Melanoma	Multiple-Dose Monotherapy	Phase I (2022)	18	Adults (18–99)	University of Florida	United States	Not yet recruiting
22	NCT05533697	Encodes IDO and PD-L1 Proteins (mRNA-4359)	In vivo	Advanced Solid Tumors	Monotherapy Combination with Immune Checkpoint Inhibitors	Phase I/II (2022)	194	Adults (≥18)	ModernaTX, Inc.	United States	Recruiting
23	NCT05359354	Encodes Neoantigens (PGV002)	In vivo	Solid Tumors	Combination with Immune Checkpoint Inhibitors	N/A (2022)	36	Adults (18–75)	YueJuan Cheng with Collaboration of Peking Union Medical College Hospital	China	Recruiting
24	NCT05456165	Encodes Neoantigens (GRT-R902)	In vivo	Colorectal Cancer	Combination with GRT-C901, Immune Checkpoint Inhibitors and Adjuvant Chemotherapy	Phase II (2022)	1	Children and Adults (≥12)	Gritstone bio, Inc.	United States	Terminated
25	NCT05227378	Encodes Neoantigens	In vivo	Gastric Cancer	Monotherapy Combination with Immune Checkpoint Inhibitors	N/A (2022)	36	Adults (18–75)	Shen Lin, Peking University	China	Not yet recruiting
26	NCT05198752	Encodes Neoantigens (SW1115C3)	In vivo	Malignant Solid Tumors	Multiple-Dose Monotherapy	Phase II (2022)	30	Adults (18–80)	Stemirna Therapeutics	United States	Recruiting
27	NCT05192460	Encodes Personalized Neoantigens (PGV002)	In vivo	Gastric Cancer Esophageal Cancer Liver Cancer	Monotherapy Combination with Immune Checkpoint Inhibitors	N/A (2022)	30	Adults (18–75)	Jianming xu, The Affiliated Hospital of the Chinese Academy of Military Medical Sciences	China	Recruiting
28	NCT04573140	Encodes LAMP Protein	In vivo	Glioblastoma	Monotherapy	Phase I (2021)	28	Adults (≥21)	University of Florida	United States	Recruiting [9]
29	NCT04911621	Encodes WT1 Protein Loaded into Autologous Monocyte-derived DCs	Ex vivo	High Grade Glioma Diffuse Intrinsic Pontine Glioma	Combination with Chemoradiation	Phase I/II (2021)	10	Children (1–17)	University Hospital, Antwerp	Belgium	Active, not recruiting
30	NCT04526899	Encodes four tumor associated antigens: NY-ESO-1, MAGE-A3, tyrosinase, and TPTE (BNT11)	In vivo	Melanoma Stage III/IV	Monotherapy Combination with Immune Checkpoint Inhibitors	Phase II (2021)	184	Adults (≥18)	BioNTech SE with collaboration of Regeneron Pharmaceuticals	United States Australia Germany Italy Poland Spain United Kingdom	Active, not recruiting
31	NCT04486378	Encodes up to 20 neoantigens (BNT122)(RO7198457)	In vivo	Colorectal Cancer	Monotherapy	Phase II (2021)	201	Adults (≥18)	BioNTech SE	United States Belgium Germany Spain	Recruiting
32	NCT04534205	Encodes two oncoproteins: E6 and E7 (BNT113)	In vivo	Head and Neck Cancer	Combination with Immune Checkpoint Inhibitors	Phase II (2021)	285	Adults (≥18)	BioNTech SE	United States Austria Belgium Canada Czechia Hungary Portugal Germany Italy Poland Spain United Kingdom	Recruiting
33	NCT03688178	Encodes LAMP Protein Loaded into Autologous DCs	Ex vivo	Glioblastoma	Combination with Chemotherapy, Tetanus-diphtheria toxoid and Varlilumab	Phase II (2020)	43	Adults (≥18)	Gary Archer Ph.D.with Collaboration of Celldex Therapeutics	United States	Recruiting
34	NCT04382898	Encodes Five Tumor associated Antigens (BNT112)	In vivo	Metastatic Prostate Cancer	Monotherapy Combination with Immune Checkpoint Inhibitors	Phase I/II (2019)	75	Adults (≥18)	BioNTech SE	United States Germany Hungary United Kingdom	Active, not recruiting [10]
35	NCT04163094	Encodes three Ovarian Tumor Associated Antigens (W_ova1 Vaccine)(BNT116)	In vivo	Ovarian Cancer	Combination with Chemotherapy	Phase I (2019)	8	Adults and Children	University Medical Center Groningen with Collaboration of BioNTech SE	Netherlands	Terminated
36	NCT03897881	Encodes Neoantigens (mRNA-4157)	In vivo	Melanoma	Combination with Immune Checkpoint Inhibitors	Phase II (2019)	257	Adults (≥18)	ModernaTX, Inc.	United States Australia	Recruiting
37	NCT03948763	Encodes Four Most Common KRAS Mutation (mRNA-5671/V941)	In vivo	Non-Small-Cell Lung Cancer Pancreatic Cancer Colorectal Cancer	Monotherapy Combination with Immune Checkpoint Inhibitors	Phase I (2019)	70	Adults (≥18)	Merck Sharp & Dohme LLC	United States Australia Hong Kong South Korea New Zealand Singapore Taiwan	Completed
38	NCT03815058	Encodes up to 20 neoantigens (BNT122)(RO7198457)	In vivo	Melanoma	Combination with Immune Checkpoint Inhibitors	Phase II (2019)	131	Adults (≥18)	Genentech, Inc. in collaboration with BioNTech SE	United States Australia Austria Belgium Germany Spain United Kingdom	Active, not recruiting [11]
39	NCT03908671	Encodes Neoantigens	In vivo	Esophageal Cancer Non-Small-Cell Lung Cancer	Monotherapy	N/A (2019)	24	Adults (18–75)	Stemirna Therapeutics	China	Recruiting
40	NCT03480152	Encodes Neoantigens (mRNA-4650)	In vivo	Melanoma Colon Cancer Gastrointestinal Cancer Genitourinary Cancer Liver Cancer	Multiple-Dose Monotherapy	Phase I/II (2018)	5	Adults (18–70)	National Cancer Institute (NCI)	United States	Terminated [12]
41	NCT03468244	Encodes Neoantigens	In vivo	Advanced Esophageal Squamous Carcinoma Gastric Cancer Pancreatic Cancer Colorectal Cancer	Multiple-Dose Monotherapy	N/A (2018)	24	Adults (18–75)	Changhai Hospital with Collaboration of Stemirna Therapeutics	China	Unknown [13]
42	NCT03548571	mRNA from Autologous Tumor Stem Cells, mRNA of survivin and mRNA of hTERT Loaded into Autologous DCs	Ex vivo	Glioblastoma	Combination with Chemoradiation	Phase II/III (2018)	60	Adults (18–70)	Oslo University Hospital	Norway	Active, not recruiting
43	NCT03164772	Encodes 6 Tumor Associated Antigens: MUC1, surviving, NY-ESO-1, 5T4, MAGE-C2 and MAGE-C1 (BI 1361849)	In vivo	Metastatic Non-small Cell Lung Cancer	Combination with Immune Checkpoint Inhibitors	Phase I/II (2017)	61	Adults (≥18)	Ludwig Institute for Cancer Research in Collaboration with Cancer Research Institute, Boehringer Ingelheim, MedImmune LLC, CureVac AG, PharmaJet, Inc.	United States	Completed
44	NCT03289962	Encodes up to 20 neoantigens (BNT122)(RO7198457)	In vivo	Solid Tumors	Monotherapy Combination with Immune Checkpoint Inhibitors	Phase I (2017)	272	Adults (≥18)	Genentech, Inc. in Collaboration with BioNTech SE	United States Belgium Canada Germany Netherlands Spain Sweden United Kingdoms	Active, not recruiting [11]
45	NCT03313778	Encodes Neoantigens (mRNA-4157)	In vivo	Solid Tumors	Multiple-Dose Monotherapy Combination with Immune Checkpoint Inhibitors	Phase I (2017)	242	Adults (≥18)	ModernaTX, Inc. in Collaboration with Merck Sharp & Dohme LLC	United States	Recruiting [14]
46	NCT02649829	Encodes WT1 Protein Loaded into Autologous DCs	Ex vivo	Malignant Pleural Mesothelioma	Combination with Chemotherapy and Surgery	Phase I/II (2017)	28	Adults (≥18)	University Hospital, Antwerp	Belgium	Active, not recruiting
47	NCT03418480	Encodes two Onco-proteins: E6 and E7 (BNT113)	In vivo	Head and Neck Cancer Cervical Cancer Penile Cancer	Multiple-Dose Monotherapy	Phase I/II (2017)	32	Adults (≥18)	University of Southampton In collaboration with BioNTech SE	United Kingdom	Active, not recruiting
48	NCT02465268	Encodes pp65-LAMP Protein Loaded into Autologous DCs	Ex vivo	Glioblastoma Malignant Glioma Astrocytoma, Grade IV	Combination with GM- CSF and Tetanus-diphtheria toxoid	Phase II (2016)	175	Adults (≥18)	Immunomic Therapeutics, Inc.	United States	Completed
49	NCT03083054	Encodes WT1 Protein Loaded into Autologous DCs	Ex vivo	Myelodysplastic Syndromes Acute Myeloid Leukemia	Multiple-Dose Monotherapy	Phase I/II (2016)	5	Adults (18–70)	University of Campinas, Brazil	Brazil	Unknown
50	NCT02808416	Encodes Tumor Antigens Loaded into Autologous DCs (PerCellVac3)	Ex vivo	Brain Metastases	Multiple-Dose Monotherapy	Phase I (2016)	10	Adults (18–65)	Guangdong 999 Brain Hospital	China	Completed
51	NCT02808364	Encodes Tumor Antigens Loaded into Autologous DCs (PerCellVac2)	Ex vivo	Glioblastoma	Multiple-Dose Monotherapy	Phase I (2016)	10	Adults (18–65)	Guangdong 999 Brain Hospital	China	Completed
52	NCT02709616	Encodes Tumor Antigens Loaded into Autologous DCs (PERCELLVAC)	Ex vivo	Glioblastoma	Multiple-Dose Monotherapy	Phase I (2016)	10	Adults (18–65)	Guangdong 999 Brain Hospital	China	Completed
53	NCT02529072	Encodes LAMP Protein Loaded into Autologous DCs	Ex vivo	Malignant Glioma Astrocytoma Glioblastoma	Combination with Immune Checkpoint Inhibitors	Phase I(2016)	6	Adults (18–80)	Gary Archer Ph.D. in Collaboration with Bristol-Myers Squibb and Duke Cancer Institute	United States	Completed [15]
54	NCT02528682	Encodes Minor Histocompatibility Antigens Loaded into Allogeneic DCs	Ex vivo	Hematological Malignancies	Multiple-Dose Monotherapy	Phase I/II (2016)	10	Adults (≥18)	Radboud University Medical Center	Netherlands	Completed
55	NCT02649582	Encodes WT1 Protein Loaded into Autologous DCs	Ex vivo	Glioblastoma	Combination with Chemoradiation	Phase I/II (2015)	20	Adults (≥18)	University Hospital, Antwerp	Belgium	Recruiting
56	NCT02366728	Encodes CMV pp65-LAMP Protein Loaded into Autologous DCs	Ex vivo	Glioblastoma Astrocytoma, Grade IV	Combination with Chemotherapy,Tetanus-diphtheria toxoid and basiliximab	Phase II (2015)	64	Adults (18–80)	Gary Archer Ph.D.	United States	Completed [16]
57	NCT02140138	Encodes 6 Tumor Associated Antigens	In vivo	Prostate Cancer	Multiple-Dose Monotherapy	Phase II (2014)	35	Adults (≥18)	CureVac AG	Germany	Terminated
58	NCT01995708	Encodes Tumor Associated Antigens CT7, MAGE-A3, and WT1 Loaded into DCs	Ex vivo	Multiple Myeloma	Combination with Autologous Stem Cell Transplantation	Phase I (2014)	28	Adults (≥18)	Memorial Sloan Kettering Cancer Center	United States	Completed [17]
59	NCT01734304	Encodes Leukemia-associated Antigens WT1, PRAME and CMVpp65 Loaded into DCs	Ex vivo	Acute Myeloid Leukemia	Monotherapy	Phase I/II (2013)	13	Adults (18–75)	Ludwig-Maximilians - University of Munich	Germany	Completed [18]
60	NCT01686334	Encodes WT1 Antigen Loaded into Autologous DCs	Ex vivo	Acute Myeloid Leukemia	Combination with Follow-up Care and Low-intensity Chemotherapy	Phase II (2012)	130	Adults (≥18)	Zwi Berneman in Collaboration with Research Foundation	Belgium	Recruiting
61	NCT01676779	A Mixture of mRNAs Encode TriMix and One of Four Melanoma Associated Antigens (gp100, tyrosinase, MAGE-A3 or MAGE-C2 fused to DC-LAMP) Loaded into Autologous DCs	Ex vivo	Malignant Melanoma Stage III/IV	Monotherapy	Phase II (2012)	88	Adults (≥18)	UZ Brussel	Belgium	Completed [19]
62	NCT01456104	Encodes Murine tyrosinase-related Peptide 2 Loaded into Autologous DCs	Ex vivo	Melanoma	Multiple-Dose Monotherapy	Phase I (2011)	9	Adults and Children	Memorial Sloan Kettering Cancer Center in Collaboration with Rockefeller University	United States	Active, not recruiting
63	NCT01446731	Encodes PSA, PAP, survivin and hTERT Antigens Loaded into Autologous DCs	Ex vivo	Prostate Cancer	Combination with Chemotherapy	Phase II (2011)	43	Adults (≥18)	Inge Marie Svane	Denmark	Completed [20]
64	NCT01291420	Encodes WT1 Protein Loaded into Autologous DCs	Ex vivo	Glioblastoma Renal Cell Carcinoma Sarcomas Breast Cancers Malignant Mesothelioma Colorectal Tumors	Multiple-Dose Monotherapy	Phase I/II (2011)	48	Adults (≥18)	University Hospital	Belgium	Completed [21]
65	NCT02285413	Encodes Tumor Associated Antigens gp100 and tyrosinase Loaded into DCs	Ex vivo	Melanoma	Monotherapy Combination with Chemotherapy	Phase II (2011)	54	Adults (18–70)	Radboud University Medical Center	Netherlands	Completed [22]
66	NCT01278914	Tumors mRNA Loaded into Autologous DCs	Ex vivo	Prostate Cancer	Monotherapy	Phase I/II (2011)	N/A	Adults (≥45)	Oslo University Hospital	Norway	Completed
67	NCT01456065	Encodes TERT Antigen (+survivin Peptide) Loaded into Autologous DCs	Ex vivo	Ovarian Epithelial Cancer	Multiple-Dose Monotherapy	Phase I (2010)	15	Adults (18–75)	Life Research Technologies GmbH	Austria Hungary	Unknown [23]
68	NCT01197625	Primary Prostate Cancer Tissue mRNA and mRNA Encodes hTERT and Survivin Loaded into Autologous DCs	Ex vivo	Prostate Cancer	Monotherapy	Phase I/II (2010)	30	Adults (18–75)	Oslo University Hospital	Norway	Active, not recruiting [24]
69	NCT01530698	Encodes gp100, tyrosinase, TLR4 and CD70 Loaded into Autologous DCs	Ex vivo	Melanoma	Monotherapy	Phase I/II (2010)	28	Adults (18–70)	Radboud University Medical Center	Netherlands	Completed [25]
70	NCT00965224	Encodes WT1 Protein Loaded into Autologous DCs	Ex vivo	Acute Myeloid Leukemia Chronic Myeloid Leukemia Multiple Myeloma	Combination with Standard Therapy	Phase II (2010)	50	Adults (≥18)	University Hospital	Belgium	Unknown [26]
71	NCT01066390	Encodes TriMix Loaded into Autologous DCs	Ex vivo	Melanoma	Multiple-Dose Monotherapy	Phase I (2009)	18	Adults (≥18)	Bart Neyns	Belgium	Completed [27]
72	NCT00978913	Encodes hTERT, survivin and p53 (if Tumors Express p53) Loaded into Autologous DCs	Ex vivo	Breast Cancer Malignant Melanoma	Combination with Cyclophosphamide	Phase I (2009)	31	Adults (≥18)	Inge Marie Svane	Denmark	Completed [28]
73	NCT00890032	Encodes Brain Tumor Stem Cells Specific Antigens Loaded into Autologous DCs	Ex vivo	Recurrent Central Nervous System Neoplasm	Multiple-Dose Monotherapy	Phase I (2009)	50	Adults (≥18)	John Sampson in Collaboration with National Cancer Institue	United States	Completed
74	NCT00846456	mRNA from Tumor Stem Cells Loaded into Autologous DCs	Ex vivo	Glioblastoma Brain Tumor	Monotherapy	Phase I/II (2009)	20	Adults (18–70)	Oslo University Hospital	Norway	Completed [29]
75	NCT00961844	mRNA for hTERT, Survivin and Tumor Derived mRNAs Loaded into Autologous DCs	Ex vivo	Metastatic Malignant Melanoma	Combination with Chemotherapy	Phase I/II (2009)	15	Adults (≥18)	Steinar Aamdal	Norway	Terminated
76	NCT00940004	Encodes gp100 and tyrosinase Loaded into Autologous DCs	Ex vivo	Melanoma	Multiple-Dose Monotherapy	Phase I/II (2009)	20	Adults (18–70)	Radboud University Medical Center	Netherlands	Completed [25]
77	NCT00929019	Encodes gp100, tyrosinase, and Loaded into Autologous DCs	Ex vivo	Uveal Melanoma	Multiple-Dose Monotherapy	Phase I/II (2009)	23	Adults (18–75)	Radboud University Medical Center	Netherlands	Terminated [30]
78	NCT00923312	Encodes 5 NSLC Antigens: New York esophageal squamous cell carcinoma-1, melanoma antigen family C1/C2, survivin, and trophoblast glycoprotein (cv9201)	In vivo	Non-Small Cell Lung Cancer (NSLC)	Multiple-Dose Monotherapy	Phase I/II (2009)	46	Adults (18–75)	CureVac AG	Germany Switzerland	Completed [31]
79	NCT01153113	Encodes hTERT Antigen Loaded into Autologous DCs	Ex vivo	Prostate Cancer	Multiple-Dose Monotherapy	Phase I/II (2008)	0	Adults (≥18)	University of Florida	United States	Withdrawn
80	NCT00626483	Encodes pp65-LAMP Protein Loaded into Autologous DCs	Ex vivo	Malignant Neoplasms Brain	Combination with Basiliximab and GM-CSF	Phase I (2007)	34	Adults (18–120)	Gary Archer Ph.D. in Collaboration with National Cancer Institute (NCI)	United States	Completed [32]
81	NCT00204516	Encodes Melanoma Associated Antigens: Melen-A1, Mage-A1, Mage-A3, surviving, GP100 and tyrosinase	In vivo	Malignant Melanoma	Combination with GM-CSF	Phase I/II (2007)	31	Adults (18–80)	University Hospital Tuebingen	Germany	Completed
82	NCT00514189	mRNA from Tumor Lysate Loaded into Autologous DCs	Ex vivo	Leukemia	Multiple-Dose Monotherapy	Phase I (2007)	2	Adults and Children	M.D. Anderson Cancer Center	United States	Terminated
83	NCT00510133	Encodes hTERT Loaded into Autologous DCs (GRNVAC1)	Ex vivo	Acute Myelogenous Leukemia	Monotherapy	Phase II (2007)	21	Adults (≥18)	Asterias Biotherapeutics, Inc.	United States	Completed [33]
84	NCT00639639	Encodes pp65-LAMP Protein Loaded into Autologous DCs	Ex vivo	Malignant Neoplasms of Brain	Combination with Tetanus Toxin and Autologous Lymphocyte Transfer	Phase I (2006)	42	Adults (≥18)	Gary Archer Ph.D. in Collaboration with National Cancer Institute	United States	Active, not recruiting [16]
85	NCT00834002	Encodes WT1 Protein Loaded into Autologous DCs	Ex vivo	Acute Myeloid Leukemia (AML)	Monotherapy	Phase I (2005)	10	Adults (≥18)	University Hospital, Antwerp	Belgium	Completed [34]
86	NCT00204607	Encodes Melanoma Associated Antigens: Melen-A1, Mage-A1, Mage-A3, surviving, GP100 and tyrosinase	In vivo	Malignant Melanoma	Combination with GM-CSF	Phase I/II (2004)	20	Adults (18–75)	University Hospital Tuebingen	Germany	Completed [35]
87	NCT00243529	Encodes gp100 and tyrosinase Loaded into Autologous DCs	Ex vivo	Melanoma Stage III or IV	Monotherapy Comparison with Peptide Vaccines	Phase I/II (2004)	64	Adults (18–75)	Radboud University Medical Center	Netherlands	Completed [36]
88	NCT00228189	Encodes Carcinoembryonic Antigen Loaded into Autologous DCs	Ex vivo	Colorectal Cancer Liver Metastases	Combination with Chemotherapy	Phase I/II (2003)	30	Adults (18–75)	Radboud University Medical Center	Netherlands	Completed [37]
89	NCT01278940	Tumors mRNA Loaded into Autologous DCs	Ex vivo	Malignant Melanoma	Combination with IL-2	Phase I/II (2002)	31	Adults (≥18)	Oslo University Hospital	Norway	Completed [38]
90	NCT00006430	Whole Tumor mRNA Loaded into Autologous DCs	Ex vivo	Prostate Cancer	Multiple-Dose Monotherapy	Phase I (2000**)**	N/A	Adults (≥18)	National Center for Research Resources (NCRR)	United States	Unknown
**Adoptive Cell Therapy**											
91	NCT05969041	Encodes a Chimeric Antigen Receptor against TROP2 for Monocytes	Ex vivo	Epithelial Tumors	Monotherapy	Phase I (2023)	48	Adults (≥18)	Myeloid Therapeutics	United States	Recruiting
92	NCT04745403	Encodes a TCR against HBV for T Cells (SAFE-T-HBV)	Ex vivo	HBV-Related Hepatocellular Carcinoma	Multiple-Dose Monotherapy	Phase I (2022)	10	Adults (21–75)	Lion TCR Pte. Ltd.	Singapore	Recruiting
93	NCT05302037	Encodes a Chimeric Antigen Receptor against NKG2DL for Allogeneic γδ T Cells	Ex vivo	Advanced Solid Tumors Hematological Malignancies	Multiple-Dose Monotherapy	Phase I (2022)	9	Adults (≥21)	CytoMed Therapeutics Pte Ltd	Singapore	Unknown
94	NCT05169489	Encodes CBLB-Targeting megaTAL Enzyme to Knockout the CBLB Gene (Part of bbT369)	Ex vivo	B Cell non-Hodgkin's lymphoma	Monotherapy	Phase I/II (2022)	50	Adults (≥18)	2seventy bio	United States	Recruiting
95	NCT05195294	Encodes a T Cell Receptor Against HBV Antigen (LioCyx-M)	Ex vivo	HBV-Related Liver Cancer	Multiple-Dose Monotherapy Combination with Lenvatinib	Phase I/II (2022)	55	Adults (18–75)	Lion TCR Pte. Ltd.	Singapore	Not yet recruiting
96	NCT04981691	Encodes Chimeric Antigen Receptor Against Mesothelin for Autologous T Cells	Ex vivo	Malignant Solid Neoplasms	Combination with Chemotherapy	Phase I (2021)	12	Adults (18–80)	UTC Therapeutics Inc.	China	Recruiting
97	NCT04816526	Encodes a Chimeric Antigen Receptor against BCMA for Autologous CD8^+^ T Cells (Descartes-08)	Ex vivo	Multiple Myeloma	Monotherapy	Phase II (2021)	30	Adults (≥18)	Cartesian Therapeutics	United States	Recruiting
98	NCT04625205	Encodes a personal neoantigen to stimulate T cells (BNT221)	Ex vivo	Melanoma	Monotherapy	Phase I (2020)	72	Adults (18–75)	BioNTech US Inc.	Belgium Netherlands Spain	Recruiting [39]
99	NCT03994705	Encodes a Chimeric Antigen Receptor against BCMA for Autologous CD8^+^ T Cells (Descartes-11)	Ex vivo	Multiple Myeloma	Combination with Chemotherapy	Phase I/II (2019)	25	Adults (≥18)	Cartesian Therapeutics	United States	Active, not recruiting
100	NCT03431311	Encodes a T Cell Receptor Against Mutant Form of TGFβRII	Ex vivo	Colorectal Cancer	Multiple-Dose Monotherapy	Phase I/II (2018)	1	Adults (≥18)	Oslo University Hospital	Norway	Terminated
101	NCT03415100	Encodes a Chimeric Antigen Receptor against NKG2DL for Autologous/Allogeneic NK Cells	Ex vivo	Solid Tumors	Combination with IL-2	Phase I (2018)	30	Adults (18–70)	The Third Affiliated Hospital of Guangzhou Medical University	China	Unknown [40]
102	NCT02315118	Encodes a Chimeric Receptor Against FCγRIII to Mediate Antibody-dependent Cell Cytotoxicity	Ex vivo	Chronic Lymphocytic Leukemia Non-Hodgkin's Lymphoma	Combination with Rituximab and IL-2	Phase I/II (2014)	18	Adults and Children (6 Months-80 Years)	National University Hospital	Singapore	Unknown [41]
103	NCT01355965	Encodes a Chimeric Antigen Receptor against mesothelin for Autologous T Cells	Ex vivo	Malignant Pleural Mesothelioma	Single and Multiple-Dose Monotherapy	Phase I (2011)	18	Adults (≥18)	University of Pennsylvania	United States	Completed [42]
**Passive Immunotherapy**											
104	NCT05262530	Encodes a Monoclonal Antibody Against CLDN6 (BNT142)	In vivo	Solid Tumors	Monotherapy	Phase I/II (2022)	288	Adults (≥18)	BioNTech SE	United States Singapore Spain	Recruiting
105	NCT04683939	Encodes a Monoclonal Antibody Against CLDN18.2 Protein (BNT141)	In vivo	CLDN18.2-positive Solid Tumors	Multiple-Dose Monotherapy Combination with Chemotherapy	Phase I/II (2022)	13	Adults (≥18)	BioNTech SE	United States Canada Spain	Terminated
106	NCT05113342	Encodes a bispecific Antibody Against BCMA and IL-12 (Descartes-25)	Ex vivo	Multiple Myeloma	Monotherapy	Phase I/IIa (2021)	20	Adults (≥18)	Cartesian Therapeutics	United States	Recruiting
**Immuno-epigenetic modulation**											
107	NCT05978102	Encodes IL2v (STI-7349)	In vivo	Advanced Solid Tumors	Multiple-Dose Monotherapy Combination with Immune Checkpoint Inhibitor	Phase I/II (2023)	124	Adults (18–75)	The Fourth Affiliated Hospital of Zhejiang University School of Medicine	China	Recruiting
108	NCT06088004	Encodes human single-chain IL-12 protein (ABO2011)	In vivo	Advanced Solid Tumors	Multiple-Dose Monotherapy	Phase I (2023)	40	Adults (≥18)	Suzhou Abogen Biosciences Co., Ltd.	China	Enrolling by invitation
109	NCT05392699	Encodes Human Single Chain IL-12 (ABOD2011)	In vivo	Advanced Solid Tumors	Single-Dose Monotherapy Multiple-Dose Monotherapy	Phase I (2022)	60	Adults (≥18)	Cancer Institute and Hospital, Chinese Academy of Medical Sciences	China	Recruiting
110	NCT05497453	Encodes Epigenetic Controller Proteins (OTX-2002)	In vivo	Liver Cancer	Multiple-Dose Monotherapy Combination with Tyrosine Kinase Inhibitors Combination with Immune Checkpoint Inhibitor	Phase I/II (2022)	190	Adults (≥18)	Omega Therapeutics	United States	Recruiting
111	NCT04710043	Encodes IL-7 and IL-2 (BNT152 and BNT153)	In vivo	Solid Tumors	Monotherapy	Phase I (2021)	170	Adults (≥18)	BioNTech SE	United States	Recruiting
112	NCT04455620	Encodes a modified IL-2 (BNT151)	In vivo	Solid Tumors	Monotherapy Combination with Anti-cancer Agents	Phase I/II (2021)	84	Adults (≥18)	BioNTech SE	United States Spain United Kingdom	Recruiting
113	NCT03946800	Encodes IL-12 (MEDI1191)	In vivo	Solid Tumors	Combination with Immune Checkpoint Inhibitors	Phase I (2019)	61	Adults (18–101)	MedImmune LLC	United States France Netherlands Spain	Completed [39,43]
114	NCT03871348	Encode cytokines: IL-12sc, IL-15sushi, IFNα and GM-CSF (BNT131) (SAR441000)	In vivo	Advanced Solid Tumors	Monotherapy Combination with Immune Checkpoint Inhibitors	Phase I (2019)	77	Adults (≥18)	Sanofi in collaboration with BioNTech RNA Pharmaceuticals GmbH	United States Belgium France Germany Netherlands Spain	Active, not recruiting [44]
115	NCT03788083	Encodes CD70, CD40L and TLR4 (TriMix)	In vivo	Breast Cancer	Monotherapy	Phase I (2018)	36	Adults (18–85)	UZ Brussel with Collaboration of eTheRNA immunotherapies	Belgium	Recruiting
116	NCT03739931	Encodes OX40L, IL-23 and IL-36γ (mRNA-2752)	In vivo	Malignant Solid Tumors Lymphoma	Monotherapy Combination with Immune Checkpoint Inhibitors	Phase I (2018)	264	Adults (≥18)	ModernaTX, Inc. with Collaboration of AstraZeneca	United States Israel	Active, Not Recruiting [45]
117	NCT03323398	Encodes OX40L Protein	In vivo	Malignant Solid Tumors Lymphoma Ovarian Cancer	Multiple-Dose Monotherapy Combination with Immune Checkpoint Inhibitors	Phase I/II (2017)	79	Adults (≥18)	ModernaTX, Inc.	United States	Terminated (Not Effective)
**Combined strategies**											
118	NCT04503278	Encodes Claudin 6 to amplify CLDN6-Specific CAR T cells (BNT211)	In vivo	Solid Tumors	Combination with CAR T Cells	Phase I/II (2020)	114	Adults (≥18)	BioNTech Cell & Gene Therapies GmbH	Germany Netherlands	Recruiting [46]
119	NCT03396575	Autologous Whole Tumor mRNA for Autologous T Cells and DCs	In vivo	Diffuse Intrinsic Pontine Glioma Brain Stem Glioma	Combination with Chemoradiation, Tetanus-diphtheria toxoid, GM-CSF and Autologous Hematopoietic Stem Cells	Phase I (2018)	21	Adults and Children (3–30)	University of Florida in Collaboration with Accelerate Brain Cancer Cure and Lyla Nsouli Foundation	United States	Recruiting
120	NCT03394937	Mixture of TriMix and 5 Tumor Associated Antigens (ECI-006)	In vivo	Melanoma	Multiple-Dose Monotherapy	Phase I (2017)	21	Adults (18–80)	eTheRNA immunotherapies	Belgium Spain	Terminated [47]
**Using mRNAs as an adjuvant for peptide vaccines**											
121	NCT02452307	mRNA/Protamin	In vivo	Prostate Cancer	Combination with Peptide Vaccine	Phase I/II (2004)	36	Adults (45–80)	University Hospital Tuebingen	Germany	Unknown

### 2.1 Therapeutic vaccination

Therapeutic vaccination is the oldest and most widely used mRNA-based anti-tumor strategy, which deals with ex-vivo or in-vivo loading of antigen-presenting cells, especially dendritic cells (DCs) with whole tumor mRNA, synthetic mRNA encoding tumor-associated antigens (TAAs) or tumor-specific antigens (TSAs) [48]. These pulsed DCs (pDCs) stimulate T lymphocytes to attack and eradicate tumors. Theoretically, mRNA-pDCs are considered more efficient than peptide-based alternatives since the cytoplasmic translation of intact antigens induces potent and extensive cytotoxic T-cell (CTL) response with less human leukocyte antigen (HLA) restriction. However, previous studies reported contradictory evidence about superiority of mRNA-pDCs over peptide alternatives as an anti-tumor agent [37,49]. Hence, more comparative studies are required to illustrate the validity of this hypothesis. Since ex-vivo-generated mRNA-pDCs have exhibited poor efficacy in clinical studies, in-vivo generation of pDCs using formulated mRNAs has become a trend in majority of recent trials (Table 1). The reason behind this shift of interest can be rationalized by the fact that in-vivo pDCs, unlike ex-vivo pDCs, encounter antigens naturally with appropriate cellular interactions/stimulations, leading to the optimum activation of CTL responses [50]. In a recent clinical study by BioNTech, prostate cancer patients were treated with BNT112 (encoding five prostate cancer TAAs) as monotherapy or in combination with immune checkpoint inhibitors (NCT04382898). Based on the results, it appeared that BNT112 is safe and induces potent anti-tumor immune responses, leading to a significant reduction in the serum levels of prostate-specific antigen (PSA) [10]. It has been demonstrated previously that designing mRNA sequences using in silico analysis enhances their stability and translational efficacy, improving immunotherapy outcomes. Cancer antigens are individually unique, which makes personalized antigen selection essential. It is crucial to utilize in silico approaches for the prediction and prioritization of best-fit candidates from the pool of tumor antigens due to the fact that various epitopes show different levels of immunogenicity [[51], [52], [53]]. As part of a study conducted by Sahin et al., somatic mutations of melanoma tumors were first organized as the mutanome and ranked according to their predicted high affinity binding to autologous HLA class II, high expression of mutation-encoding RNA, and predicted binding to HLA class I. The selected Neo-epitopes induced T-cell responses against melanoma tumors and significantly prolonged progression-free survival [54]. Another way to improve mRNA cancer vaccine efficacy is to modify their structural features. A key component of this strategy involves enhancing the presence of antigen-encoding mRNA in the cytosol by deleting nuclear localization signals and redirecting peptides to the endoplasmic reticulum and endosomal/lysosomal compartments. It has been demonstrated that these modifications improve translational efficacy and antigen presentation on major histocompatibility complex I (MHCI), which in turn improves the response of CTLs to selected antigens [55]. Codon-optimized mRNA sequences with a higher GC content and suboptimal codon usage have also shown enhanced translational efficacy in vitro and in vivo [[56], [57], [58]].

### 2.2 Adoptive cell therapy

The delivery of manipulated or natural immune cells, such as tumor-infiltrating lymphocytes (TILs), autologous/allogeneic T cells, Natural killer (NK) cells, and macrophages into the patients' body for the destruction of cancer cells is a sophisticated immunotherapeutic approach known as adoptive cell therapy [59]. Viral-transduced CAR-T cells have demonstrated astonishing results in clinical trials performed on hematological malignancies and are now available as FDA-approved treatment options. However, life-threatening adverse events, such as cytokine release syndrome (CRS) and immune effector cell-associated neurotoxicity syndrome (ICANS), hinder their complete success [60]. Due to the transient expression of CARs, mRNA-transfected CAR-T cells can result in better treatment process management and reduce the rate of side effects [61]. After successful preclinical evaluations, BNT211 (an unmodified CLDN6 mRNA for enhancing persistence of anti- CLDN6 CAR T cells) was recently entered into a phase I/II trial for solid tumors (NCT04503278). Phase I data have demonstrated acceptable safety and relative efficacy, especially for patients with limited treatment options [46].

According to the literature, CAR-NK cells are considered a safer alternative compared to CAR-T cells due to different cytokine secretion profiles and slower expansion rates [62,63]. In a recent phase I clinical trial performed in China, patients with metastatic colorectal cancer received mRNA-transfected CAR NK cells against NKG2D ligand in combination with IL-2 (NCT03415100). The generation of ascites, tumor burden in ascites samples, and the viability of NKG2DL-expressing tumors were decreased without any grades ≥3 adverse effects [40]. The use of CARs in macrophages to eliminate tumors by phagocytosis is an innovative approach that has recently entered clinical trials for breast cancer (NCT04660929). Although mRNA-based alternatives have not yet entered into human studies, they have demonstrated incentive preclinical results [64].

### 2.3 Humoral immunotherapy

Although many FDA-approved monoclonal antibodies are used as effective immunotherapy options for the treating solid tumors and blood malignancies, they are only partially unchallenged. The quality control process is often costly because of the risk of viral contamination, mutations occurring in antibody-producing mammalian cell lines, and improper post-translational modifications. Furthermore, the short half-life due to rapid plasma clearance and digestion by serum enzymes demands frequent injections, adding to the high cost of treatment for patients [65]. mRNA technology can be harnessed as an alternative to overcome many of these challenges. The need for mammalian cell lines and their associated problems can be eliminated by delivering formulated mRNAs that turn the patients' bodies into a bioreactor for antibody production. mRNAs not only steadily yield high amounts of proteins but also reduce sudden exposure, injection-dosing frequency, and their related side effects. In addition, mRNA therapeutics could be more affordable for patients due to their rapid, flexible, and cost-effective manufacture. After many encouraging preclinical studies using mRNA-based passive immunotherapy to treat various types of cancer, such as intestinal and breast cancer, BioNTech Company has just launched two phases (I/IIa) of clinical trials using this strategy [66,67]. Monoclonal antibodies against Claudin 18.2 (NCT04683939) and Claudin 6 (NCT05262530) are utilized to destroy cancer cells via the antibody-dependent cell-cytotoxicity mechanism in patients with solid tumors. These trials aim to evaluate safety, pharmacokinetics, and dose escalation. Descartes-25 (a tumor-tropic allogeneic mesenchymal stem cell transfected with an mRNA encoding bispecific antibody against BCMA and IL-12) has been designed by Cartesian Therapeutics, Inc. as the world-first off-the-shelf mRNA/Stem cell medicine. The safety and efficacy (local delivery bispecific antibody and concentration of anti-tumor IL-12 around cancer cells) will be evaluated in a currently launched phase I/IIa for multiple myeloma patients (NCT05113342).

### 2.4 Immuno-epigenetic modulation

In this strategy, mRNAs are engaged to deliver epigenetic controllers, cytokines, and Immune receptors/ligands for suppressing oncogenes, stimulating the immune system, and igniting the tumor microenvironment. Epigenetic modulation via mRNAs has opened new horizons in cancer treatment. Research on OTX-2002, the first mRNA therapeutic developed by the Omega Company, revealed exclusive downregulation of the MYC gene in tumors, inhibition of tumor development, and induction of apoptosis [68,69]. OTX-2002 encodes two epigenetic controllers targeting the MYC. Recently, the Omega Company has conducted a phase I/II clinical trial to evaluate OTX-2002 monotherapy or in combination with immune checkpoint inhibitors or tyrosine kinase inhibitors in liver cancer patients (NCT05497453). This trial will evaluate the safety, tolerability, pharmacokinetics, pharmacodynamics, and anti-tumor activity of OTX-2002. The application of mRNAs encoding immune modulators, such as cytokines and immune receptors or ligands, is now being investigated in numerous clinical trials [70]. Lately, mRNA-2752 (encoding OX40L, IL-23, and IL-36γ) has been used by Moderna/AstraZeneca to treat 23 patients with solid tumors as monotherapy or in combination with Durvalumab (NCT03739931). mRNA-2752 was tolerable and effective as treated patients showed tumor suppression, and the plasma levels of pro-inflammatory cytokines, such as IFN-γ and TNF-α, were significantly increased [45].

## 3 mRNA therapeutics in treatment of autoimmune diseases

**Fig. 2:**
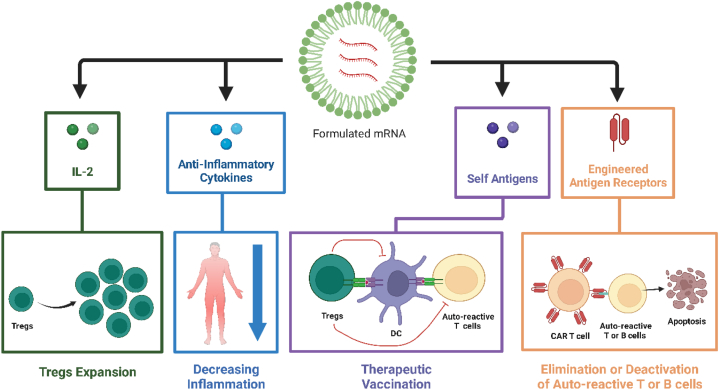
mRNA-based strategies for the treatment of autoimmune diseases.

Human society is challenged more than ever by autoimmune diseases (ADs). Many efforts have been made to treat ADs with the primary goal of managing self-reacting T and B lymphocytes [71]. Several mRNA-based strategies have shown reassuring results in preclinical studies, including therapeutic vaccination using self-antigens in the absence of co-stimulatory signals, elimination of auto-reactive lymphocytes using engineered antigen receptors, expansion of regulatory T cells, and decreasing inflammation with anti-inflammatory cytokines (Fig. 2).

Recently, the Moderna Company has entered a regulatory T cells (Tregs) expansion strategy using Interleukin-2 (IL-2) mRNA into the clinical phase. While effector T cells and NK cells express a low-affinity dimeric IL-2 Receptor (IL2R), Tregs exclusively express a high-affinity heterotrimeric IL2R [72]. Several studies have shown that low doses of IL-2 can mainly induce the expansion of Tregs, immune tolerance, and reduction of disease severity [72,73]. Although low-dose IL-2 therapy has encouraging results in preclinical studies and clinical trials, the presence of some challenges, such as rapid clearance from body fluids and unspecific activation of non-Tregs, have hindered the full effectiveness of this approach. The Moderna Company has tried to overcome these obstacles by using mRNA technology. mRNA-6231 encodes a mutated version of IL2R (high affinity to Tregs IL2R and fewer off-targets) fused to serum albumin (enhanced half-life and longer persistence in body fluids). A phase I clinical trial has just launched to investigate the safety, pharmacokinetics, pharmacodynamics, and tolerability of single or multiple doses in healthy adults (NCT04916431). If successful, this mRNA therapeutic will revolutionize the treatment of many types of ADs. On its website, Moderna has also announced that other mRNA pharmaceuticals are under preclinical development, including mRNA-6981 (encodes PD-L1 checkpoint blockade and reduces auto-reactivity of lymphocytes) for ADs, such as autoimmune hepatitis, and will enter into clinical evaluation in the near future. The world premiere mRNA-based CAR-T cell for the autoimmune disease has been designed by Cartesian Therapeutics, Inc. and entered phase Ib/IIa to cure Generalized Myasthenia Gravis (NCT04146051). Descartes-08 (mRNA-transfected CAR-T cell against BCMA) aims to destroy the auto-reactive B lymphocytes, reducing the concentration of auto-antibodies in the patient's body.

## 4 mRNA therapeutics in treatment of infectious diseases

The recent COVID-19 (coronavirus disease-2019) pandemic revealed that, compared to other platforms, mRNA possesses significant potential to combat infections. period One of the biggest advantages of mRNA technology is its great capability to be modified and updated into a new version in a short time to deal with emerging variants and limit the outbreak, especially in pandemic conditions with the high mutational burden. Within weeks of identifying the gene sequences of new or mutated viruses, mRNA vaccines can be synthetically produced. A research team at Imperial College London developed a COVID-19 vaccine candidate just two weeks after sequencing the SARS-CoV-2 virus [74]. Moderna announced on its website that the sequence of the mRNA-1273 vaccine candidate was finalized two days after disclosing the virus's genomic sequence, and the first clinical batch of mRNA-1273 was completed after only about 27 days. In addition to rapid and cost-effective development, the innate-adjuvanticity properties of mRNA molecules can excellently stimulate the immune system and enhance antigen delivery to T and B cells. Even though mRNA technology has proven its efficacy and practical value in a global battlefield during the COVID-19 pandemic, its long-term effects on the human body remain unclear. A low rate of acute side effects has been reported so far for the short-term use of mRNAs; however, understanding the long-term effects is necessary to unlock their full potential and enable widespread use [75,76]. Some of the COVID-19 mRNA vaccines are listed in Table 2.

**Table 2:** mRNA vaccines developed for COVID-19 in clinical trials studies.

	NCT Number	Name	Target	Phase	Results
1	NCT04368728	BNT162b1	SARS-CoV-2 spike protein receptor–binding domain (RBD)	I/II	It induces strong cellular and humoral responses but causes more systemic reactogenicity than BNT162b2. So BNT162b2 has been chosen for further studies [77,78].
2	NCT04368728	BNT162b2	SARS-CoV-2 full-length spike	III	Two doses (30 μg per dose, given 21 days) are effective about 95% in preventing infection in adults. It induces strong humoral and cellular responses [77,79,80].
3	NCT04283461 NCT04405076 NCT04649151 NCT04470427	mRNA-1273	pre-fusion SARS-CoV-2 spike protein	III	It was the first vaccine that entered into the clinical trial. Phase III clinical trial indicates that two doses are effective about 94% in preventing infection and effective in the prevention of severe disease [81,82].
4	NCT04668339 NCT04480957	LUNAR® -COV19 ARCT-021	SARS-CoV-2 full length spike glycoprotein	II	It is a self-transcribing and replicating RNA (STARR) vaccine. Only one dose induced humoral and cellular responses in mice properly. Compared with conventional mRNA vaccines, these vaccines caused more and longer expression (till seven days instead of 1 day) and therefore caused more innate and B cells and T cells responses [83].
5	NCT04515147 NCT04449276	CVnCoV	A stabilized form of SARS-CoV-2 S protein	I	Two doses given 28 days induce proper immune responses and do not cause any adverse complications. 12 μg dose was chosen for phase II and III clinical trials [84].
6	NCT04566276	ChulaCoV19	SARS-CoV-2 Transmembrane spike	I/II	Induced well tolerated, dose-dependent, B cell, and T cell response. Following the second dose, pain, fever, chills, fatigue, and headache have been seen commonly.

The advent of mRNA-based therapeutics has significantly expanded the scope of available therapies. For instance, Epstein-Barr virus (EBV) infection now has a vaccine candidate (mRNA-1189, NCT05164094) containing four mRNAs that encode proteins involved in virus entry into cells. This vaccine aims to prevent infectious mononucleosis and control symptoms. Phase I of this study is estimated to be completed in 2023.

In general, mRNA vaccines used in research and industry could be divided into two groups.

### 4.1 Self-amplifying RNA or replicon RNA

These mRNAs encode antigens of interest and viral replication machinery. Therefore, RNA amplification occurs intracellular, and antigen expression is increased. HIV (human immunodeficiency virus), Rabies, and COVID-19 are three viral diseases with self-amplifying vaccine candidates that have entered clinical trials.

#### 4.1.1 HIV, the hot topic among the mRNA vaccines

mRNA vaccines have already proven successful against COVID-19, and HIV might be the next major obstacle to overcome. Standard antiviral treatments have several drawbacks, including ineffectiveness, high costs, and unwanted side effects [85]. Several HIV vaccines have been created and are now in clinical trials, intending to prevent infection by optimizing the antigen expression of the virus in the body. For instance, eODGT8 is a nanoparticle that carries mRNA-1644, an RNA replicon designed to mimic the binding site of the HIV envelope and activate specific progenitor B cells to produce broadly neutralizing antibodies against the virus. The first trial on it (NCT03547245), which ended in 2021, had promising results, and now two other ongoing trials with some modification on eODGT8 are recruiting. (NCT05001373 and NCT05414786). HIV has other types of mRNA vaccines that will be discussed later in this article.

#### 4.1.2 Rabies

Following promising results in the preclinical phase [86], mRNA-based vaccines for Rabies have been introduced in clinical trials. GSK3903133A is a self-amplifying RNA vaccine against Rabies (NCT04062669) that encodes glycoprotein G and has been examined in term of immunogenicity and safety in 82 patients (No result has been provided yet).

#### 4.1.3 COVID-19

Among numerous vaccines developed for COVID-19 and studied comprehensively in clinical trials, a few belong to the replicon mRNA. A study (ISRCTN17072692) examined whether the antibody that was induced by the vaccine was sufficient to block or limit virus entry and reduce the risk of subsequent infection. The researchers were guided by the hypothesis that low doses of the vaccine would cause an increase in the spike protein expression and immune responses. Self-amplifying RNA offers potential advantages over non-amplifying RNA, including the ability to achieve an equivalent adaptive immune response with a lower dosage (NCT05012943) [87]. If comparing the doses of the currently available two SARS-CoV-2 mRNA vaccines by Moderna (mRNA-1273) and BioNTech/Pfizer (BNT162b2) that are 100 μg and 30 μg, respectively, in a clinical trial (Phase 3) on sa-RNA vaccine (ARCT-154) against COVID-19 by Arcturus Therapeutics, only 5 μg sa-RNA are used (NCT05012943). Consequently, sa-RNA has the potential to be used as a single-dose regimen, potentially reducing non-responder rates and alleviating the burden of drug manufacturing, all while maintaining comparable efficacy to mRNA-based approaches. Therefore, it might represent a promising and potentially superior option in the epidemic situation [88].

### 4.2 Non-replicating mRNA

These mRNAs solely encode the desired antigens. There are two ways to use in-vitro transcribed mRNAs (IVT) as vaccinations; the first step is the electroporation of DCs *ex-vivo* with the desired mRNA and then re-inoculate them into the host [88]. The second approach is to inoculate directly IVT mRNAs that express particular antigens *in-vivo*.

#### 4.2.1 Dendritic cell mRNA vaccines

In the case of DC mRNA vaccines, patients are immunized with DCs transfected with IVT mRNA encoding antigens of infectious organisms. As previously mentioned, antigen-encoding mRNA is translated inside the cytoplasm of DCs, and antigenic peptides processed by MHC class I are presented on CD8 + T cells. Among infectious diseases, the dendritic cell mRNA vaccine has mainly focused on finding a therapeutic candidate for HIV-1. The majority of clinical trials on DC mRNA vaccines investigate dendritic cells electroporated with mRNAs encoding various HIV-1 antigens called AGS-004 (NCT00672191, NCT01069809, NCT02042248, and NCT00381212). Safety and tolerability as well as treatment efficacy (especially after termination of antiretroviral therapy) have been under extensive studies since 2006.

Along with self-amplifying vaccines, a study designed in 2009 by Gandhi et al. showed that autologous DCs transfected with HIV-1 Gag and Nef mRNA (NCT00833781) were well tolerated and did not have severe side effects. Patients in this study also received a DC vaccine loaded with keyhole limpet hemocyanin (KLH) to assess the possibility of immune response induction against a neo-antigen. Even though de novo CD4^+^ and CD8^+^ T-cells proliferation against neo-antigen increased, the response was transient, and no interferon-gamma ELISPOT response was observed. Overall, vaccination cannot significantly enhance the viral elimination process. Due to the limited sample size of 15 cases, it is difficult to explore the underlying causes. However, hypotheses such as malfunction of HIV-derived DCs [89], improper support cocktail (in this research, TNF-α, IL-1β, IL-6, and prostaglandin E2), and activation of T regulation have been proposed [90].

#### 4.2.2 Direct injection of non-replicating mRNA vaccine

This technique of vaccine manufacture, which was developed as a result of investigations conducted in 1990, is quick and easy. In this method, the IVT mRNAs directly release the target antigen via many routes. So far, this strategy has been used for several diseases, and investigations on RNA modification and nanoparticle carrier choices are being conducted in quest of the best effective vaccine formulation [91].

Vaccine mRNA CV7201 (NCT02241135) encodes the rabies virus glycoprotein. In the first phase of the clinical trial, 101 participants experienced three serious adverse events, but only one (Bell's palsy) was related to the vaccination. CV7201 has an excellent tolerability profile and generates cellular and humoral responses, according to published studies. However, immune responses to lethal rabies infection seem weak, and this vaccine requires additional development. For instance, the immunogenicity of CV7201 is highly dependent on the injection method. This problem led to the development of a potential mRNA vaccine made with lipid nanoparticles (LNPs), CV7202 (NCT03713086). The most recent study demonstrated that the moderate dosage of this vaccination elicited enough rabies-neutralizing antibodies and fulfilled WHO standards. However, patients may have a prophylactic response at larger dosages [92].

##### 4.2.2.1 Influenza

Among mRNA vaccines for Influenza, mRNA-1440 (NCT03076385) and mRNA-1851 (NCT03345043) were the first mRNA vaccines encoding full-length hemagglutinin (HA) glycoprotein sequences. These studies concluded that vaccines were safe and without any serious adverse events. In contrast to the adequate humoral immune responses against H10N8 and H7N9 influenza viruses, no acceptable cellular immunity responses were detected, showing that HA antigen is not a promising antigen for T cells [93]. However, the efficacy, immunogenicity, and safety of HIV-related mRNA drugs are still being intensively investigated by several vaccine candidates in multiple clinical trials, some of which are listed in Table 3.

**Table 3:** Some mRNA-therapeutics candidates investigating in trials.^a^

	NCT Number	Name	Title	Phase	Status	Sponsors/Collaborators
1	NCT04956575	mRNA-1010	A Study of mRNA-1010 Seasonal Influenza Vaccine in Healthy Adults	Phase 1/2	Completed	ModernaTX, Inc.
2	NCT05415462	mRNA-1010	A Study of mRNA-1010 Seasonal Influenza Vaccine in Adults	Phase 3	Completed	ModernaTX, Inc.
3	NCT05333289	mRNA-1020 mRNA-1030	A Study of mRNA-1020 and mRNA-1030 Seasonal Influenza Vaccines in Healthy Adults	Phase 1/2	Completed	ModernaTX, Inc.
4	NCT05426174	mRNA NA Vaccine	Phase I, Randomized, Modified Double-blind, Parallel-group, Active-controlled, Multi-arm, Dose-escalation Study to Assess the Safety and Immunogenicity of Monovalent mRNA NA Vaccine in Adult Participants 18 Years of Age and Older	Phase 1	Active, Not recruiting	Sanofi Pasteur
5	NCT05252338	CVSQIV	A Study to Evaluate the Safety, Reactogenicity and Immunogenicity of Vaccine CVSQIV in Healthy Adults	Phase 1	Completed	CureVac/GlaxoSmithKline
6	NCT03076385	VAL-506440	Safety, Tolerability, and Immunogenicity of VAL-506440 in Healthy Adult Subjects	Phase 1	Completed	ModernaTX, Inc.
7	NCT03345043	VAL-339851	Safety, Tolerability, and Immunogenicity of VAL-339851 in Healthy Adult Subjects	Phase 1	Completed	ModernaTX, Inc.
8	NCT05375833	mRNA-1073 mRNA-1010 mRNA-1273	A Safety, Reactogenicity, and Immunogenicity Study of mRNA-1073 (COVID-19/Influenza) Vaccine in Adults 18–75 Years Old	Phase 1/2	Completed	ModernaTX, Inc.
9	NCT05446740	GSK4382276A	A Study on the Safety, Reactogenicity and Immune Response of a Vaccine Against Influenza in Healthy Younger and Older Adults	Phase 1	Recruiting	GlaxoSmithKline/CureVac

a Some results may be available in the financial section of the Moderna's website (https://investors.modernatx.com/news/news-details/2022/Moderna-Reports-Fourth-Quarter-and-Fiscal-Year-2021-Financial-Results-and-Provides-Business-Updates/default.aspx.

##### 4.2.2.2 Zika

Clinical studies have shown that influenza-like Zika virus infection may be preventable with mRNA-based vaccines. ModernaTX, Inc. produced mRNA-1893, a vaccine that encodes the structural proteins of the Zika virus, and it was able to generate neutralizing antibodies in its initial phase I study (NCT04064905). Phase II launched in 2021 (NCT04917861), with mRNA-1893 being studied in over 800 people.

##### 4.2.2.3 RSV

Respiratory syncytial virus (RSV) is another respiratory infection that can be a potential clinical target for mRNA drugs. There are three current active clinical trials at phases 1 to 3 evaluating the tolerability, safety, and immunogenicity of mRNA-1345, an RSV vaccine candidate, with ModernaTX leading the way. It encodes an engineered F protein sequence, a highly conserved protein involved in viral fusion with the cell membrane, to establish a stable protein complex (NCT04528719, NCT05127434, and NCT05330975).

##### 4.2.2.4 CMV

A vaccine candidate, mRNA-1647, is currently undergoing several clinical trial studies for cytomegalovirus (CMV) infection. This vaccine encodes several immunogenic parts of the virus particle (such as glycoprotein B) and can prevent infection in epithelial cells. In addition to safety (phase 3, NCT05085366), the optimal vaccine dose is still being determined in clinical trials (NCT04232280).

### 4.3 Efficacy of mRNA vaccines in elderly and immunedeficient patients

Even though mRNA vaccines have been used in cancer for two decades, they seem to be a safe and successful medicinal choice. Decades of follow-up and monitoring of any adverse effects, as well as examination for potential incompatibilities with other pharmacological and clinical situations, are necessary. In the same way that previous trials were conducted, current research is looking at the effectiveness of mRNA vaccines for COVID-19 (mRNA-1273 and BNT162b2) in elderly and immunocompromised individuals who do not meet the criteria for participation in vaccine trials. Recent studies have demonstrated the high effectiveness of Pfizer and Moderna mRNA vaccines in preventing COVID-19 infection among the elderly and reducing the risk of hospitalization [94,95]. Despite the overall positive impact of mRNA vaccines on immunocompromised patients, the exact immunologic response to vaccination varies among individuals with different immunodeficiency conditions. In patients with HIV-related immunodeficiency, vaccination with mRNA vaccines against COVID-19 has been found to be safe and immunogenic [96]. Conversely, a study has shown that in individuals with functional B-cell defects, cellular immune response was stimulated more effectively than humoral immunity [97]. In another study, most patients with primary immunodeficiency exhibited a favorable humoral immune response, while some lacked an adequate cellular response [98]. These findings underscore the need for further research to comprehensively understand the impact of COVID-19 vaccines on individuals with diverse immunodeficiency conditions.

The investigations into alternative vaccination for individuals with cancer and autoimmune diseases are of significant importance due to their strong desire for it. These case studies have been documented in Table 4.

**Table 4:** COVID-19 mRNA vaccines investigated in cancer and autoimmune diseases.

	Trial Number	Title	mRNA vaccine	Indication	Status	Details
1	NCT05050461	Immune Response After SARS-CoV-2 Vaccination in a Context of Non-Hodgkin Lymphoma (LYMPHO-CoVac)	mRNA-1273 or BNT162b2	Non-hodgkin Lymphoma	Unknown	Check for modification of immune response after vaccination in patients with anti-CD20 monoclonal antibody immunotherapy.
2	NCT04872738	Patient Experiences With the COVID-19 Vaccination After Breast Cancer Treatment (LymphVAX)	mRNA-1273 or BNT162b2	Breast Cancer-Related Lymphedema	Active, Not Recruiting	Whether vaccination could impart Lymphedema; due to node swelling, which is a common side effect of mRNA vaccines.
3	NCT04951323	Impact of the Immune System on Response to Anti-Coronavirus Disease 19 (COVID-19) Vaccine in Allogeneic Stem Cell Recipients (Covid Vaccine Allo)	BNT162b2	Hematopoietic Neoplasms	Recruiting	Check for development of protective immune response against COVID in allo-hematopoietic cell transplantation recipients.
4	NCT05028374	COVID-19 VAX Booster Dosing in Patients With Hematologic Malignancies (Multiple Myeloma AL Amyloidosis Chronic Lymphocytic Leukemia)	mRNA-1273	Hematologic Malignancies	Completed	The trial will assess the antibody level following booster by the background of a low amount of antibodies against coronavirus in these patients.
5	NCT04862806	Safety, Efficacy of BNT162b2 mRNA Vaccine in CLL	BNT162b2	Chronic Lymphocytic Leukemia	Unknown	Check for the safety and efficacy of the vaccine.
6	NCT04969601	Anti-Covid-19 Vaccine in Children With Acute Leukemia and Their Siblings (PACIFIC)	BNT162b2	12 to 15 children with acute leukemia	Unknown	Check for the safety and efficacy of the vaccine.
7	NCT04792567	Exploring the Immune Response to SARS-CoV-2 modRNA Vaccines in Patients With Secondary Progressive Multiple Sclerosis (AMA-VACC) (AMA-VACC)	mRNA-1273 or BNT162b2	Secondary Progressive Multiple Sclerosis	Completed	By having their adaptive immune cell trapped in lymph nodes following standard treatment of MS, the vaccine's efficacy needs to be figured out.

### 4.4 Safety of mRNA-based vaccines

While the mRNA-based vaccines have emerged as a pivotal tool to control the COVID-19 pandemic and prevented numerous mortality and hospitalizations, recent studies revealed that they might not be as risk-free as they seem. Given the novel foundation upon which these vaccines are built, the post-vaccination effects may be critical in weighing the danger of vaccination against the risk of no vaccine. A probable link between mRNA-based vaccinations (Pfizer-BioNTech or Moderna) and the increased risk of cardiovascular disorders, such as myocarditis and pericarditis, has been documented. The incidence of post-vaccination COVID-19 mRNA is significantly higher than that previously reported after smallpox vaccination. Most of the reported cases were middle-aged men with or without a history of cardiovascular disease who presented with mild symptoms that occurred mostly three days (1–13 days) after the second dose and were treated with conventional therapy [99]. Nevertheless, according to some case studies [100], the number of adolescent males being diagnosed is on the rise, and no mortality has been documented as of yet. Despite the existence of hypotheses, the precise underlying process is yet unclear. Additionally, a study in Israel found that the rate of Bell's palsy following BNT162b2 mRNA COVID-19 vaccination appears to be higher than expected during clinical trials [101].

In contrast, the overall rate found with influenza vaccinations and other viral vaccines is lower, according to the WHO pharmacovigilance database. Bell's palsy may develop after the first or second dosage of the vaccination. Older women are the most often affected; while, with the right prognosis and prompt treatment, a recovery rate of up to 90% is achievable [101]. Overall, the risks of the two previously described side effects of mRNA-based vaccinations are judged to be modest, and both studies have limitations (such as lack of generalizability). Therefore, determining the possible long-term consequences and the effect of clinical history on the occurrence of these side effects requires further investigations.

### 4.5 mRNA-based passive immunization

The delayed development of adaptive immunity can pose challenges in certain situations, such as when fatal or incurable infections occur in individuals without prior exposure. In these cases, the disease can progress to a critical stage before adaptive immunity is established, leading to a lethal or incurable phenotype. However, immediate immunological control of infectious agents upon exposure can prevent the disease from reaching a critical stage. Additionally, passive immunity can be employed as a temporary strategy to confer immunity against infectious agents in immunocompromised patients. Notably, mRNA technology has shown promise in developing passive immunity against HIV and Rabies viruses. Preclinical studies by Pardi et al. and Thran et al. demonstrated the successful protection of a single administration of LNP-encapsulated mRNA molecules encoding neutralizing antibodies against HIV, rabies, and botulinum toxin in mice models [102,103]. These findings highlight the potential of mRNAs as a clinically efficient therapeutic for passive immunity-related prophylaxis and treatment of various infections.

## 5 mRNA therapeutics in gene therapy

### 5.1 Genetic disorders

mRNA therapy can offer unique solutions as an effective treatment for monogenic diseases. To date, most treatments are based on gene therapy and modification. Although this method provides significant results, it might have risks and adverse effects that prompt researchers to search for better ways to reduce the complications of these diseases. Among the promising alternatives, mRNA therapy allows transient expression of the desired proteins in target cells, despite the continuous protein expression in gene therapy, and at the same time, has longer-term effects than protein drugs. The use of mRNA components of nuclease proteins, such as ZFNs, TALENs, and Cas9, as gene-editing tools, enables a shorter presence of nucleases in cells and prevents damage to undesirable sequences [104,105]. As an example, in Refractory Viral Keratitis, a rare severe infection of HSV (Herpes Simplex Virus), mRNA of CRISPR/Cas9 was delivered to the cornea of 3 patients to disrupt the virus causing the stromal keratitis (NCT04560790). The corresponding mRNA was expressed in virus-like particles (VLPs); however, no specific results have been published yet. NTLA-2001, an mRNA-based gene therapy utilizing CRISPR technology, has recently progressed to the clinical phase. This therapy involves the administration of LNP-encapsulated mRNA encoding cas9 protein and gRNA targeting misfolded transthyretin (TTR), a protein predominantly accumulated in the heart and nerves of transthyretin amyloidosis patients. Following successful outcomes in preclinical studies, a phase 1 clinical trial was conducted on six transthyretin amyloidosis patients to assess the safety and pharmacodynamics of a single-dose injection. The results demonstrated mild side effects and a significant dose-dependent reduction in serum TTR protein levels [106].

An additional advantage of mRNA therapy over gene therapy is the absence of sequence (DNA) integration into the host cell genome and the reduced possibility of mutagenicity and cancer in target cells. Due to the chronic nature of monogenic disorders, multiple medication injections are necessary, which limit the use of viral vectors (immunity to the vector prevents re-administration) [107].

In pioneering mRNA-based gene therapy, granulocyte cells from patients with chronic granulomatous disease were collected by apheresis and returned to the patient's blood after being corrected by mRNA encoding NADPH oxidase. This research (NCT05189925) began in 2022 and is presently in the first phase of a clinical trial. Initial findings indicate that this treatment is well-tolerated and exerts a substantial impact on the population of NADPH Oxidase-containing cells even 72 h post-administration of the mRNA therapeutic [108]. Familial hypercholesterolemia is another genetic condition in the first phase of the clinical trial. The intervention is composed of mRNA combined with nanoparticles in exosomes carrying the low-density lipoprotein receptor (LDLR) (NCT05043181). In response to the expression of functional receptors, LDL uptake may be increased, resulting in a decrease in blood cholesterol levels. The findings may be encouraging since they may eliminate the need for liver transplantation in patients with the homozygous form of this disease.

Preclinical studies have investigated the therapeutic efficacy of mRNA for a range of genetic disorders in which treatment options are limited or unavailable, such as metabolic diseases [[109], [110], [111]]. Preclinical investigations utilizing mRNA-based therapy for life-threatening arginase deficiency, caused by a mutated ARG1 gene, revealed successful expression of liver-targeted LNP-encapsulated mRNA encoding ARG1 in an arginase deficiency mouse model. The treatment demonstrated tolerability, improved urea cycle activity, enhanced liver arginase activity, and exhibited no hepatotoxicity [111]. In another preclinical study targeting the genetic modification of hematopoietic stem cells carrying the sickle cell anemia genetic defect, LNP-formulated mRNA encoding the editing system effectively targeted the stem cell marker CD117 and corrected hematopoietic sickle cells in vivo [112]. The mRNA-based drug for ornithine transcarbamylase (OTC) deficiency (ARTC810) completed its phase I clinical trial in 2020 (NCT04416126), and phase II is currently underway (NCT05526066). Similarly, other metabolic disorders, such as methylmalonic acidemia (NCT05295433), propionic acidemia (NCT05130437), and glycogen storage disease type 1a (NCT05095727), which are therapeutic candidates for mRNA-based drugs, have recently entered clinical stages.

### 5.2 Cellular reprogramming

Although mRNA drugs are mainly known for cancer vaccines and infectious diseases, many studies have shown their high potential in the treatment of a wide range of disorders and the development of a new approach, such as cell reprogramming. Synthetic mRNAs encoding pluripotency factors can directly reprogram differentiated cells into embryonic-like stem cells through multiple transfections. The generation of mRNA-derived induced pluripotent stem cells (iPSCs) from human somatic cells is under various studies to discover the most efficient and rapid transformation protocol [113].

Moreover, allergen-encoding mRNA vaccines are the most favorable candidates for treating type 1 allergy. Even limited priming by mRNA in infants provides long-term immunity due to the natural exposure to allergens which act as a booster for the vaccination [114].

In addition to mRNA vaccines, mRNA-based therapies for heart failure and myocardial infarction have also entered clinical trials. Cardiac progenitor cells and local fibroblasts contribute to activation through paracrine factors to induce regeneration in the heart muscle and reduce fibrosis at the repair site. Modified mRNAs encoding IGF-1 [115], VEGF-A [116], and mutant FSTL1 [117] have undergone preclinical studies, and there are two ongoing clinical trials for VEGF-A (NCT03370887, NCT02935712). The latter helps to form more blood vessels and heal diabetic wounds [118].

## 6 Conclusion and future perspectives

COVID-19 infection laid the groundwork for the mRNA platform. However, a significant focus is being directed at utilizing mRNA's intrinsic immunity-activation feature, combined with high protein expression, to combat localized neoplastic tumors. Unlike viruses with common antigens that allow a single sequence to vaccinate all individuals in a population, cancer has a personalized bias. As a result, finding appropriate antigens is difficult, which is possibly to be the reason why cancer mRNA vaccines have not been approved. A single neo-antigen may trigger sufficient immune reactions in one patient, but it is considered non-immunogenic in another and may even cause autoimmunity. Furthermore, another implication of mRNA therapeutics appears to be the possibility of acting as a vehicle for gene editing. In contrast to infectious diseases and cancer, the immune-stimulatory properties of the mRNA platform should reach the lowest level possible in gene therapy approaches. It has been shown that LNP, a conventional nucleic acid formulation used in the COVID-19 vaccine, is subject to inflammatory reactions that may impair its efficacy [119]. For the gene therapy approach to be successful, further progress in the mRNA platform is required, especially in the development of non-immunogenic formulations and targeted delivery methods.

It appears that further investigation is necessary to develop better carriers for multiple injection treatments due to inflammatory reactions triggered by the injection of an LNP-formulated mRNA vaccine. In the case of LNP, preclinical studies would be less valuable since model animals release much higher concentrations of cytokines than humans [120]. Additional studies are needed to investigate whether repeated dosing can lead to lipid accumulation in target or off-target tissues [121]. The attraction of mRNA-based therapies lies in their safety and ability to be manufactured in large quantities in a cell-free environment in a short period for clinical applications. The protein synthesis occurs in patients' bodies instead of in complex bioreactors, which are more prone to error. Further, gene therapy applications greatly benefit from tight regulation of protein expression in cells. To expand the range of diseases and conditions covered by mRNA therapeutics, formulation technology and in vivo delivery systems must progress step by step. Enhancing endosomal escape remains an important goal in improving protein production efficiency [122,123].

Innovation will lead to novel and highly effective mRNA-based therapies, awaiting vaccines against other infectious diseases such as COVID-19, cancers, autoimmune diseases, and potentially neurodegenerative diseases as well. An LNP-formulated mRNA encoding granulocyte-macrophage colony-stimulating factor (GM-CSF) was found to induce a neuroprotective effect via increased Treg populations in Parkinson's disease animal models [124]. Based on the findings from these studies, mRNA therapy can pave the way for innovative strategies in addressing previously untreatable conditions. Additionally, mRNA technology can economically revolutionize the medical industry. The agility of the mRNA platform makes it a viable option for personalized medicine-based therapies, which are a global trend nowadays. The emergence of Contract Development and Manufacturing Organizations (CDMOs) has enabled the large-scale manufacturing of mRNA-based drugs. Moreover, previous studies have demonstrated that mRNA pharmaceuticals can be distributed without cold chain logistics, eliminating many costly processes in drug transport [125]. Therefore, mRNA therapeutics have the potential to significantly reduce medication costs and facilitate drug distribution and patient access in various healthcare systems, especially in developing countries.

## Funding

None.

## Data availability statement

Data referenced in article.

## CRediT authorship contribution statement

**Roham Deyhimfar:** Writing – original draft. **Mehrnaz Izady:** Writing – original draft. **Mohammadreza Shoghi:** Writing – original draft. **Mohammad Hossein Kazazi:** Writing – original draft. **Zahra Fakhraei Ghazvini:** Writing – original draft. **Hojjatollah Nazari:** Visualization. **Zahra Fekrirad:** Writing – review & editing, Conceptualization. **Ehsan Arefian:** Writing – review & editing, Conceptualization.

## Declaration of competing interest

The authors declare that they have no known competing financial interests or personal relationships that could have appeared to influence the work reported in this paper.
